# Chemical Mechanisms of Cerebral Ischemia–Reperfusion Injury and Neuroprotection in the Thrombectomy Era

**DOI:** 10.3390/ijms27219733

**Published:** 2026-10-30

**Authors:** Leonidas D. Panos, Dib Chakif

**Affiliations:** 1 Department of Neurology, School of Medicine, Democritus University of Thrace, University General Hospital of Alexandroupolis, 68100 Alexandroupolis, Greece; 2 Department of Chemistry, Biochemistry and Pharmaceutical Sciences, University of Bern, 3012 Bern, Switzerland

**Keywords:** ischemic stroke, cerebral ischemia-reperfusion injury, mechanical thrombectomy, mitochondrial dysfunction, oxidative stress, ferroptosis, neuroprotection

## Abstract

Mechanical thrombectomy has significantly improved outcomes for patients with large-vessel occlusion stroke. However, restoring the artery does not always re-establish full microvascular blood flow, tissue rescue, or a good neurological outcome. Ischemia–reperfusion injury varies over time, with distinct biochemical mechanisms dominating during ischemia, early reperfusion, and the subsequent hours. During ischemia, factors like adenosine triphosphate (ATP) depletion, ionic imbalance, calcium overload, mitochondrial dysfunction, acidosis, and metabolic changes such as succinate build-up render brain tissue highly vulnerable. When blood flow resumes, oxygen delivery to this stressed tissue can induce rapid mitochondrial redox shifts, electron leakage, reactive oxygen and nitrogen species formation, iron-dependent radical generation, and lipid peroxidation. Damage may then persist for hours through ongoing mitochondrial dysfunction, phospholipid hydroperoxide accumulation, reactive aldehydes, ferroptosis, blood–brain barrier breakdown, microvascular issues, and inflammation. Much of the mechanistic evidence supporting these processes derives from experimental studies, and their relevance to clinical outcomes after thrombectomy requires further validation. This narrative review tracks these chemical changes across the main phases of cerebral ischemia–reperfusion injury and connects them to modern thrombectomy practices. It also explores how the timing of each process may inform neuroprotective strategies, drug delivery methods, patient selection via imaging, and future clinical trial approaches.

## 1. Introduction

Acute ischemic stroke remains a leading cause of death and long-term disability worldwide [1], despite significant advances in prevention, stroke-unit care, intravenous thrombolysis, and endovascular procedures. At the tissue level, focal cerebral ischemia rapidly triggers a complex chemical and metabolic crisis. When blood flow ceases, the brain is deprived of oxygen and glucose. As a result, oxidative phosphorylation declines, redox balance shifts, acid-base homeostasis is disrupted, ion gradients collapse, and mitochondria and membrane lipids become more vulnerable to damage. Restoring blood flow is crucial, but it also initiates a new phase of biochemical changes. The sudden return of oxygen, nutrients, ions, inflammatory cells, and redox-active molecules to tissue that has already undergone significant alterations during ischemia can cause additional injury, known as cerebral ischemia–reperfusion injury [2,3].

These mechanisms have gained increasing importance with the advent of thrombectomy. Mechanical thrombectomy has revolutionized the management of large-vessel occlusion and can significantly reduce disability in appropriate patients [4,5,6,7,8,9]. However, timing remains crucial; the longer the delay to achieve reperfusion, the lower the likelihood of a favorable outcome, as viable tissue continues to be lost even after the artery is reopened [10]. Additionally, thrombectomy has highlighted another issue: angiographic recanalization does not necessarily equate to biological tissue recovery. Reopening the large artery does not guarantee complete capillary perfusion, preservation of the neurovascular unit, or neurological improvement [11,12,13,14].

Poor outcome after recanalization has several possible causes: irreversible injury before treatment, persistent downstream ischemia, distal embolization, procedural complications, edema, hemorrhagic transformation, and systemic disturbances. Reperfusion-induced injury refers specifically to additional damage caused or amplified by restoration of flow and reoxygenation; deterioration after reopening an artery does not by itself establish this causal mechanism [2,11,12,15]. Persistent ischemia, distal embolization, and incomplete microvascular reperfusion primarily reflect ongoing impairment of oxygen and substrate delivery, whereas reperfusion-induced injury involves mechanisms directly triggered or amplified by reoxygenation, including mitochondrial ROS (reactive oxygen species) generation and oxidative damage. Mitochondrial dysfunction and inflammatory responses may originate during ischemia and become exacerbated following reperfusion, and therefore should not be considered exclusively reperfusion-dependent processes. Similarly, edema, hemorrhagic transformation, and delayed infarct expansion represent multifactorial secondary consequences rather than direct evidence of reperfusion-induced injury. In this review, experimental no-reflow denotes failure of capillary perfusion despite restoration of upstream patency. Clinical studies generally use persistent tissue hypoperfusion on CT or MR perfusion as an operational surrogate, rather than directly observing capillary obstruction [11,12,13,14]. Angiographic reperfusion grades assess downstream territorial filling and do not resolve individual capillaries. Residual arterial occlusion, distal emboli, reocclusion, perfusion delay, edema-related compression, and established infarction must therefore be considered before attributing a perfusion deficit to capillary no-reflow. Experimental contributors include pericyte constriction, endothelial swelling, platelet–leukocyte interactions, microthrombi, and impaired red-cell passage [16,17,18,19,20,21,22]. These may coexist with reoxygenation injury but are not interchangeable with it. Macrovascular recanalization, microvascular reperfusion, and cellular metabolic recovery are distinct, interacting levels of recovery.

The interval between recanalization and tissue recovery also shapes neuroprotective strategies. Many drugs that reduced infarct size in experimental models failed in clinical trials because of issues such as delayed treatment after irreversible damage, uncertain reperfusion outcomes, widely varying stroke mechanisms, poor drug delivery to ischemic areas, neglecting patient age and comorbidities, and overemphasizing a single neuronal pathway [23,24,25,26,27,28]. Thrombectomy helps address some of these challenges by enabling real-time confirmation of recanalization, allowing treatment timing around reperfusion, providing direct access for local drug delivery, and using imaging to identify potentially salvageable tissue [15]. Consequently, neuroprotection can complement reperfusion efforts, supporting tissue recovery rather than replacing reperfusion.

Candidate biochemical targets vary over time and overlap; most pathway-specific evidence discussed here is experimental. During ischemia, tissues experience energy failure, anaerobic glycolysis, acidosis, redox imbalance, loss of ion gradients, excitotoxic calcium overload, mitochondrial depolarization, accumulation of reducing equivalents, alterations in tricarboxylic acid (TCA) cycle metabolites, and weakened antioxidant defenses [2]. Once reperfusion begins, oxygen reenters the disrupted metabolic environment. This triggers rapid metabolite oxidation, mitochondrial re-energization, electron leakage, nicotinamide adenine dinucleotide phosphate (NADPH) oxidase activation, nitric oxide interactions, and iron-dependent radical formation, causing a sudden increase in oxidative and nitrosative stress [29,30]. In the following hours, lipid hydroperoxides and reactive aldehydes can accumulate, glutathione defenses may decline, iron regulation may remain disrupted, and ongoing mitochondrial damage, ferroptosis, and inflammation can continue to harm the tissue even after artery reopening [31,32,33].

Treatment timing must be evaluated against both pathway activity and residual reversibility. Preserving energy metabolism during occlusion, limiting mitochondrial oxidant production at reoxygenation, and suppressing lipid-radical propagation after flow restoration are different experimental objectives [29,30,32,33]. However, biochemical persistence alone does not demonstrate that a drug remains effective when started later. Proposed post-recanalization windows for ferroptosis or aldehyde-directed treatment require delayed-start experiments, adequate brain exposure, and ultimately randomized clinical testing.

**Figure 1:**
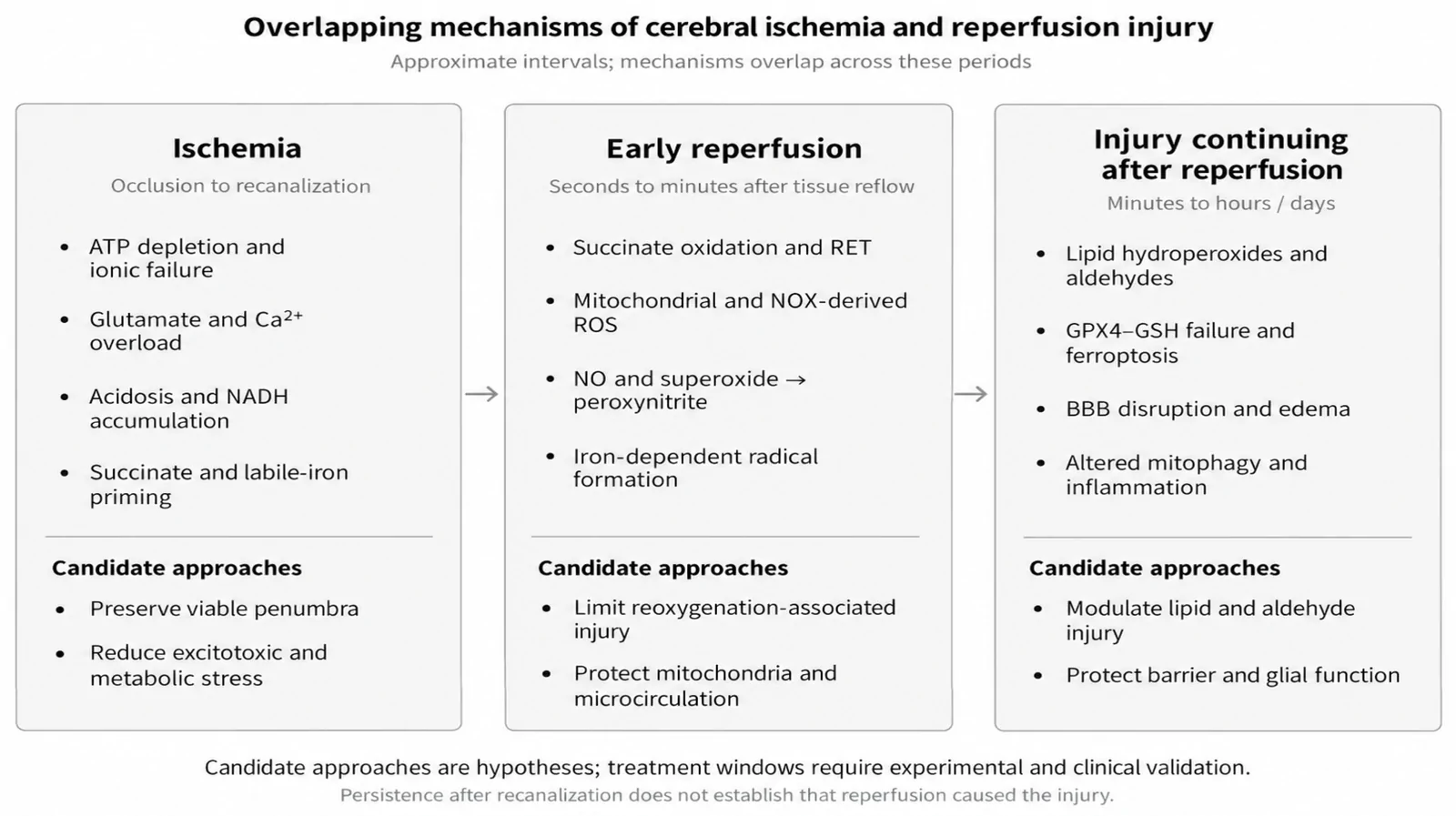
Phase-resolved model of cerebral ischemia–reperfusion chemistry and mechanism-matched neuroprotection. The dominant biochemical state changes rapidly at reperfusion, making treatment timing a mechanistic variable rather than a purely logistical one.

**Figure 2:**
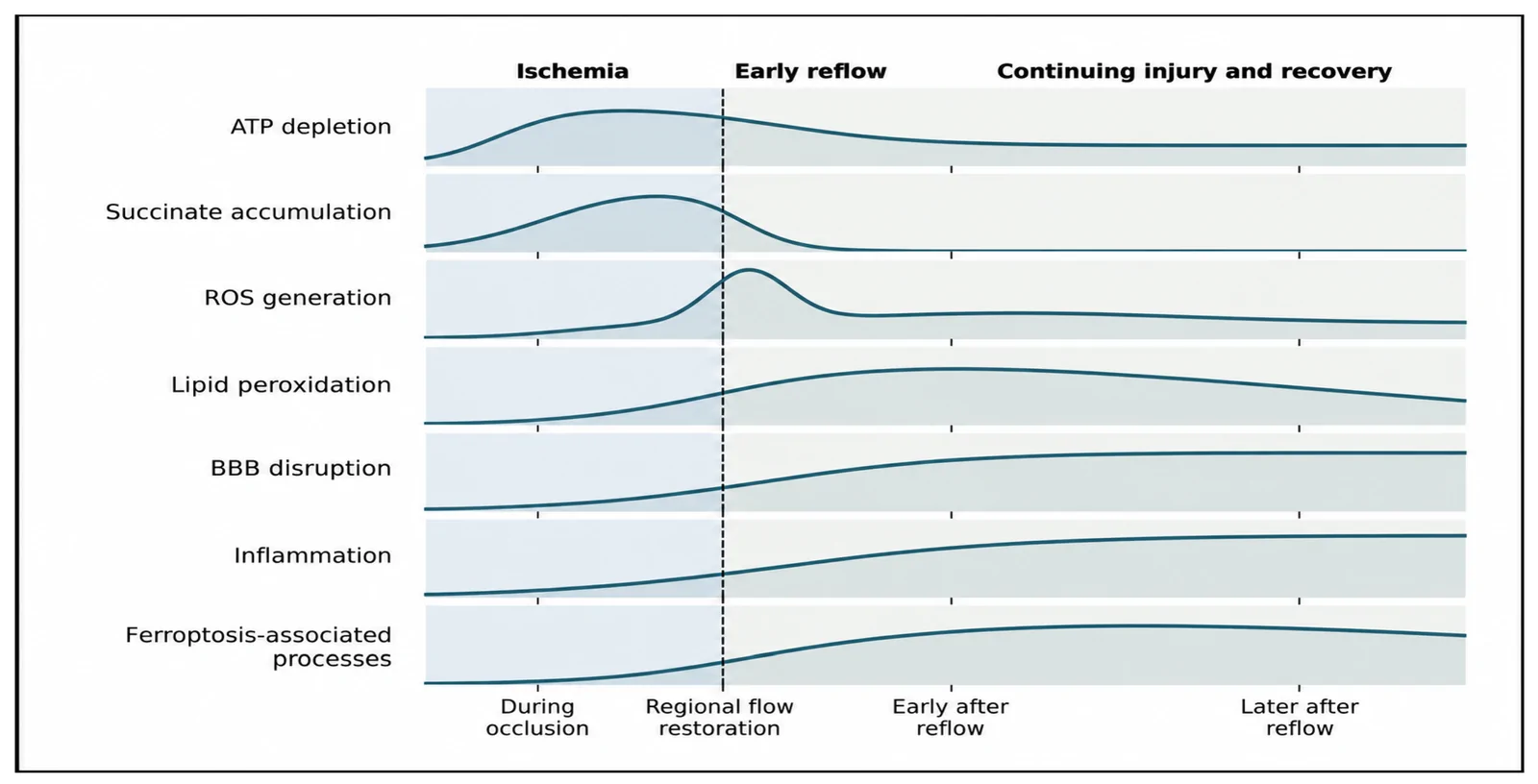
Overlapping trajectories of ischemic and post-reperfusion processes. Curves are qualitative schematics, not measured kinetics, effect sizes, or validated treatment windows. ATP depletion, lipid oxidation, barrier dysfunction, and inflammation can begin before recanalization and persist afterward; ferroptosis-associated processes are not restricted to a late phase. Reoxygenation-associated ROS may peak early in experimental settings, while other sources persist. The dashed line marks restoration of flow to a tissue region, which may differ from angiographic recanalization. Adapted conceptually from the mechanisms discussed in Section 3, Section 4 and Section 5.

This review organizes the evidence into overlapping temporal domains: ischemia, early reperfusion, and injury continuing after reperfusion. Its contribution is an integrative translational framework connecting redox chemistry to the practical constraints of thrombectomy, including drug arrival at the target, delivery route, thrombolytic co-treatment, and outcome assessment [2,15]. We distinguish experimentally supported mechanisms from human observations and randomized therapeutic evidence, and reperfusion enhancement from cytoprotection. The framework addresses mitochondrial bioenergetics, reactive oxygen and nitrogen species (ROS and RNS), iron chemistry, lipid oxidation, aldehydes, glutathione peroxidase 4 (GPX4), and ferroptosis. Table 1, Figure 1 and Figure 2 summarize the overlapping domains.

## 2. Literature Sources and Narrative Synthesis

This narrative review examines cerebral ischemia–reperfusion chemistry, neurovascular injury, and adjunctive treatment in the thrombectomy setting. The literature search for the original manuscript was conducted in August 2026 using PubMed. The review covered English-language publications from 1984 through August 2026. Relevant terminology included “Brain Ischemia”, “ischemic stroke”, “ischaemic stroke”, “cerebral ischemia”, and “cerebral ischaemia”. The thematic scope comprised six groups: (i) succinate and reverse electron transport; (ii) ferroptosis, ferritinophagy, GPX4, glutathione, acyl-CoA synthetase long-chain family member 4 (ACSL4), nuclear receptor coactivator 4 (NCOA4), and delayed treatment; (iii) aldehyde dehydrogenase 2 (ALDH2), and reactive aldehydes; (iv) no-reflow, pericytes, and reperfusion; (v) thrombectomy and endovascular treatment, including nerinetide, edaravone, hyperoxia, intra-arterial therapy, and randomized trials; and (vi) system xc^−^, cystine/glutamate exchange, excitatory amino acid transporter 3 (EAAT3), and excitatory amino acid carrier 1 (EAAC1).

Only English-language papers were included. The review incorporated animal studies, cellular experiments, human observational studies, and randomized clinical trials; human ex vivo experiments contributed mechanistic evidence. Reviews, methodological papers, and older foundational reports provided background and citation context. No formal, prespecified inclusion or exclusion criteria were applied. Publication selection was purposive and based on relevance to the biochemical mechanisms, neurovascular consequences, and therapeutic questions addressed in the review. Both authors participated in selecting and evaluating the publications. Screening was not conducted independently in duplicate, and no formal procedure for resolving disagreements was applied. Titles and available abstracts informed the assessment, and primary publications were consulted to verify central mechanistic claims, clinical effect estimates, and safety findings.

The reference list was checked for duplicate DOI entries. Multiple reports from the same randomized trial were linked by trial identity and interpreted as reports of one cohort. Animal and cellular findings were weighed according to model, cell type, ischemic severity, treatment timing, and convergence of genetic, biochemical, and pharmacological evidence. Observational studies supported human associations, whereas randomized trials were prioritized for regimen-specific efficacy and safety, considering eligibility, thrombolytic exposure, achieved reperfusion, dose, endpoints, and adverse events. The synthesis was qualitative, without quantitative pooling, a PRISMA flow diagram, a standardized risk-of-bias score, or formal certainty grading.

### Evidence Classification

Evidence is described according to its source rather than combined into a single hierarchy: (i) established clinical observations, such as the distinction between arterial patency and tissue recovery; (ii) human observational or biomarker associations; (iii) randomized clinical evidence for a specified intervention and outcome; (iv) animal-model evidence; (v) cellular, isolated-mitochondrial, or ex vivo tissue evidence; and (vi) hypothesis-generating therapeutic concepts. Human ex vivo tissue is classified as experimental, not clinical validation. Mechanistic credibility is strengthened by convergent genetic, biochemical, and pharmacological results, whereas a clinical trial establishes the effect of its tested regimen without necessarily identifying the mediating molecular pathway. These labels describe provenance and inference, not a formal GRADE assessment.

## 3. Ischemic Phase

### 3.1. Chemical and Biochemical Changes

An arterial blockage immediately causes a mismatch between energy needs and substrate supply. Neurons require substantial amounts of ATP to sustain processes like the Na^+^/K^+^-ATPase, Ca^2+^-ATPases, vesicle cycling, axonal transport, neurotransmitter reuptake, and maintaining ion gradients across membranes [2,3]. The brain has very limited oxygen storage and small carbohydrate reserves. When blood flow drops below the level needed for oxidative phosphorylation, mitochondrial ATP synthesis declines within minutes. Although phosphocreatine and remaining oxidative metabolism can briefly fill the gap, these energy reserves are quickly depleted in severely hypoperfused tissue.

As oxygen levels decrease, electron transfer to molecular oxygen at cytochrome c oxidase (complex IV) becomes less efficient. This leads to a more reduced state of electron carriers prior to complex IV, an increase in the mitochondrial NADH/NAD^+^ ratio, and diminished capacity of the TCA cycle to regenerate oxidized cofactors. Consequently, glucose metabolism shifts toward anaerobic glycolysis. Since glycolysis generates significantly less ATP than oxidative phosphorylation, pyruvate is increasingly converted into lactate to regenerate cytosolic NAD^+^ and sustain glycolysis [2,34]. The buildup of lactate and protons causes intracellular and extracellular acidosis, which further affects protein protonation, enzyme activity, ion channels, transporter functions, protein interactions, and the behavior of redox-active molecules.

As ATP levels decline, ATP-dependent ion pumps start to malfunction. This leads to Na^+^ buildup inside cells and K^+^ outside, causing neurons and glia to depolarize. Water moves into cells following Na^+^, resulting in cytotoxic swelling. Depolarization triggers voltage-gated Ca^2+^ channels and increases glutamate release. Severe ionic imbalance also impairs astrocytic glutamate uptake via excitatory amino-acid transporters, which may even reverse their function, raising extracellular glutamate levels. This excess glutamate activates N-methyl-D-aspartate (NMDA) and α-amino-3-hydroxy-5-methyl-4-isoxazolepropionic acid (AMPA) receptors, causing additional Na^+^ and Ca^2+^ influx [35,36]. The cycle of excitotoxicity directly connects energy depletion with ionic injury.

Calcium overload causes damage not only by disrupting osmotic balance but also by activating enzymes that degrade cellular components. Elevated cytosolic Ca^2+^ stimulates enzymes such as calpains, phospholipases, proteases, endonucleases, and nitric oxide synthases. For instance, cytosolic phospholipase A2 releases arachidonic acid and other polyunsaturated fatty acids from membrane phospholipids. These fatty acids can then be processed by cyclooxygenases, lipoxygenases, and cytochrome P450 enzymes or undergo nonenzymatic oxidation [36,37]. Thus, during ischemia, calcium overload increases the availability of lipid substrates that can be oxidized once oxygen supply is restored.

Mitochondria buffer calcium but also suffer damage from it. Ca^2+^ enters the mitochondrial matrix via the calcium uniporter and initially stimulates dehydrogenases [38]. However, prolonged overload causes mitochondria to depolarize, increasing the likelihood of permeability transition and further reducing ATP production [39,40]. Ischemia affects respiratory-chain proteins, shifting complex I between active and inactive states. This complex is particularly sensitive to conformational changes, redox shifts, flavin handling, and thiol chemistry during ischemia and reperfusion [34]. In severe cases, electron transport and proton pumping slow down enough for the proton-motive force to collapse. ATP synthase may then operate in reverse, consuming ATP to maintain membrane potential and depleting the remaining ATP pool.

Ischemia alters metabolite flux and can lead to succinate accumulation. Reverse succinate dehydrogenase activity, anaplerotic pathways, amino acid metabolism, purine-linked metabolism, and metabolite transport can contribute differently across tissues and experimental conditions [29,30,41,42]. Mottahedin et al. demonstrated time-dependent succinate accumulation in human and mouse brain subjected to ischemia ex vivo, and regional accumulation in a mouse focal-ischemia model [30]. The human component was an ex vivo tissue experiment, not a study of patients undergoing thrombectomy. Protection with malonate at reperfusion was demonstrated in mice. Succinate can supply electrons to ubiquinone through complex II. Reverse electron transport (RET) at complex I requires a sufficiently reduced ubiquinone pool and a high proton-motive force; its contribution changes with ischemic duration, substrate mixtures, oxygen availability, pH, calcium, respiratory-chain integrity, and mitochondrial re-energization [29,43]. This evidence arises from isolated mitochondria and controlled ischemia–reperfusion models. RET is therefore an important candidate source of early ROS. Forward electron leak at complexes I and III, NADPH oxidases, xanthine oxidoreductase, and uncoupled nitric oxide synthase can also contribute. Their relative importance must be determined for the relevant tissue, cell type, and time point [2,30].

Purine nucleotide breakdown also influences the redox state during ischemia. ATP is broken down into ADP and AMP, with AMP subsequently converted into adenosine, inosine, hypoxanthine, and xanthine. These metabolites can serve as signals and later act as substrates for oxidant-generating reactions during reperfusion. Simultaneously, the NAD^+^/NADH ratio shifts toward NADH, NADPH is utilized in metabolic and antioxidant processes, and glutathione recycling becomes more difficult [2]. This presents a paradox: ischemic cells contain highly reduced electron carriers but may have reduced capacity to neutralize peroxides upon oxygen restoration.

Iron handling also changes during ischemia. Most cellular iron is normally bound within ferritin, heme proteins, iron–sulfur clusters, and enzymes. Acidosis, lysosomal dysfunction, heme breakdown, and damage to iron-containing proteins can increase the labile pool; ferritinophagy may contribute in experimental models. Ferrous iron catalyzes the Fenton reaction in the presence of hydrogen peroxide: Fe^2+^ + H_2_O_2_ → Fe^3+^ + OH^−^ + •OH. The hydroxyl radical reacts close to its site of formation, making the location of redox-active iron important [31,32,33,44,45]. This chemistry can promote oxidative injury but does not, by itself, establish ferroptotic cell death.

### 3.2. Neurological Consequences

The neurological impact of ischemia largely depends on the residual blood flow in each tissue area. In the ischemic core, flow is insufficient to sustain ATP-dependent membrane functions, leading to rapid anoxic depolarization. The surrounding penumbra still receives some oxygen and nutrients via collateral vessels, which can delay irreversible damage. However, the penumbra is not normal tissue; it operates near energy failure, with decreased protein synthesis, disrupted ion balance, heightened excitatory activity, and increasing mitochondrial stress [46,47,48,49,50,51,52]. The likelihood of its survival primarily depends on the severity and duration of hypoperfusion and the effectiveness of collateral blood flow.

Excitotoxicity occurs when energy failure disrupts normal neurotransmission. As extracellular glutamate levels increase, NMDA receptors activate, allowing Ca^2+^ ions to enter neurons. This process also triggers neuronal nitric oxide synthase activity. While nitric oxide at normal levels aids vascular signaling, excessive amounts can be harmful in environments rich in oxidants. When nitric oxide reacts with superoxide, it produces peroxynitrite, a highly reactive nitrogen species. Even before reperfusion, calcium-dependent proteases and phospholipases damage the cytoskeleton, membranes, and organelles [16,35,36]. Concurrently, Na^+^, Cl^−^, and water influx into cells cause cytotoxic edema in neurons, astrocytes, and endothelial cells.

Different cell types respond differently to ischemia. Neurons are especially vulnerable due to their high energy needs, numerous excitatory receptors, and limited tolerance for sustained ionic changes. Astrocytes, with their enhanced glycolytic capacity and glycogen reserves, can temporarily buffer extracellular glutamate and K^+^, but severe ischemia eventually hampers these functions. Oligodendrocytes and myelinated axons are prone to glutamate toxicity, oxidative stress, and ionic imbalance. Endothelial cells and pericytes face hypoxia, acidosis, and altered shear forces, which can influence reperfusion [2,53,54]. Microglia detect ATP release, danger signals, ionic shifts, and tissue damage, triggering the subsequent inflammatory response.

Ischemic duration and collateral perfusion influence tissue survival at recanalization. Clinical imaging trials establish that selected patients can benefit from thrombectomy beyond conventional early windows and, in specific populations, despite a large infarct core [46,47,48,49,50,51,52]. These findings do not directly measure mitochondrial function, antioxidant reserve, or susceptibility to ferroptosis. Relationships between imaging-defined tissue states and such biochemical vulnerabilities remain hypotheses, as discussed in Section 6.2.

### 3.3. Potential Neuroprotective Targets

During ischemia, candidate cytoprotective treatments aim to preserve threatened tissue until flow can be restored. Hypothermia reduces metabolic demand experimentally, but its implementation, systemic effects, and clinical efficacy require separate evaluation [55]. Normobaric hyperoxia has a preclinical rationale of increasing oxygen availability through residual flow. In OPENS-2, it improved the 90-day modified Rankin Scale (mRS) distribution, with an adjusted common odds ratio of 1.65 (95% CI 1.09–2.50) [56]. A smaller late-window phase IIb trial improved its early neurological endpoint but did not demonstrate a statistically significant 90-day mRS shift [57]. These trials test oxygen delivery strategies; they do not establish inhibition of a specific redox pathway. Oxygen dose, exposure duration, selection, and the possibility of oxidative or pulmonary harm remain relevant.

Mitochondrial metabolism is a key focus. Reducing succinate buildup during ischemia or slowing its oxidation during reperfusion may decrease RET-related ROS production. In a mouse focal-ischemia model designed to approximate rapid mechanical reperfusion, administering systemic malonate at reperfusion lowered brain damage in a dose-dependent way. This supports the potential for metabolic treatments timed precisely to vessel reopening [30]. An effective drug would need to act swiftly, maintain predictable plasma levels, have manageable systemic effects, and reach the ischemic microcirculation without excessively impairing mitochondrial energy production after reperfusion.

Calcium overload links excitotoxicity to proteolysis, phospholipid breakdown, and mitochondrial injury. Broad inhibition of glutamate or calcium signaling can interfere with physiological function. Nerinetide instead targets postsynaptic density protein 95 (PSD-95)-dependent signaling downstream of NMDA receptors [36,58]. Its clinical results must be separated from the biological plausibility of this pathway: a neutral trial tests a particular intervention, dose, timing, and population, rather than proving or excluding the entire mechanism [59]. Section 6.6 examines competing explanations for failed translation.

A practical neuroprotective treatment administered before reperfusion must possess several key features. It should be safe to use before confirming the final diagnosis and reperfusion status, act within minutes, prevent hypotension or decreased collateral flow, be compatible with alteplase or tenecteplase, and ideally protect more than just neurons [15]. The aim is not to instantly normalize all cellular chemistry but to preserve threatened tissue long enough for mechanical reperfusion.

## 4. Early Reperfusion Phase

### 4.1. Chemical and Biochemical Changes

Reoxygenation changes the redox environment of ischemic tissue, but its onset and completeness vary spatially. Restoration of oxygen, substrate supply, and pH can occur in mitochondria with altered respiratory-chain function, calcium loading, and depleted antioxidant defenses [2,29]. These early changes overlap with ongoing ischemia in poorly perfused regions and with later inflammatory and lipid-mediated injury; they are not a discrete stage shared synchronously by all cells.

Succinate oxidation exemplifies this transition. During ischemia, succinate builds up and is quickly oxidized by succinate dehydrogenase (complex II) upon oxygen restoration. This process transfers electrons to ubiquinone, pushing the CoQ pool towards its reduced form, ubiquinol (CoQH_2_). If the proton-motive force is reestablished while the CoQ pool remains highly reduced, electrons can flow backward through complex I, producing superoxide via reverse electron transport [29,41,43]. Mitochondria are a significant source of early oxidative stress, but not the sole one. NADPH oxidases in endothelial cells, leukocytes, microglia, and neurons also produce superoxide from oxygen. Xanthine oxidoreductase may also contribute in certain cells and species [60,61]. Damaged mitochondria can generate ROS at complexes I and III, as well as through dehydrogenases and altered flavoproteins. While ROS are sometimes beneficial, low levels are involved in normal signaling and repair. The issue arises from high local oxidant flux, overwhelming antioxidant defenses and damaging proteins, nucleic acids, lipids, and cofactors. Effective treatment must therefore restrict harmful oxidation while preserving normal redox signaling.

Reactive nitrogen species contribute to this damage. During ischemia, calcium buildup activates neuronal nitric oxide synthase, and during reperfusion and inflammation, endothelial and inducible nitric oxide synthases are also involved. Nitric oxide quickly reacts with superoxide to produce peroxynitrite (ONOO^−^). This compound can oxidize thiols, promote lipid oxidation, nitrate tyrosine residues, damage mitochondrial proteins, and generate secondary radicals. Additionally, peroxynitrite can impair mitochondrial respiration and cause electron leakage [16,60,62,63]. These interactions explain why blocking a single source of reactive oxygen species (ROS) might be ineffective if nitric oxide production and mitochondrial dysfunction persist.

When oxygen is available again, the formation of iron-dependent radicals becomes more significant. Reperfused mitochondria and oxidases produce hydrogen peroxide, which is less reactive than superoxide but can cross membranes and persist long enough to reach redox-active iron. This ferrous iron then catalyzes the formation of hydroxyl radicals via Fenton chemistry. Reducing agents can revert iron to its ferrous form, sustaining the process. Since hydroxyl radicals react almost instantly, their damage is highly localized, affecting nearby membranes, DNA, or respiratory proteins [31,33]. Consequently, the specific location and size of the labile iron pool may influence damage more than the overall tissue iron content.

Membrane lipids are initial targets because the brain contains abundant polyunsaturated fatty acids (PUFAs). Radicals readily remove their bis-allylic hydrogens, initiating lipid peroxidation. A carbon-centered lipid radical (L•) forms first, then quickly reacts with O_2_ to create a lipid peroxyl radical (LOO•). This radical extracts a hydrogen from a neighboring PUFA, producing a lipid hydroperoxide (LOOH) and another lipid radical. This chain reaction continues: LH + X• → L• + XH; L• + O_2_ → LOO•; LOO• + LH → LOOH + L• [31,64,65]. Alpha-tocopherol interrupts the chain by donating hydrogen to lipid peroxyl radicals, while GPX4 reduces phospholipid hydroperoxides via glutathione.

During ischemia, activation of phospholipases releases arachidonic and adrenic acids and alters membrane composition. These lipids may be enzymatically oxidized by lipoxygenases and cyclooxygenases or nonenzymatically by radicals. Cardiolipin, abundant in mitochondrial membranes, is especially important because it supports normal respiratory chain structure. When oxidized, cardiolipin can destabilize respiratory supercomplexes, change cristae, promote cytochrome c release, and worsen mitochondrial dysfunction [66]. Lipid oxidation is therefore more than just an indicator of ROS exposure; it can also amplify mitochondrial damage and increase ROS production.

The mitochondrial permeability transition pore sits at the intersection of several of these processes. During reperfusion, pH normalizes, mitochondrial membrane potential rises, and mitochondria remain exposed to high matrix Ca^2+^ and oxidative stress. Together, these changes favor permeability transition. Pore opening causes loss of membrane potential, matrix swelling, outer-membrane injury, ATP depletion, and release of pro-death factors. ROS and permeability transition can reinforce each other: oxidants make pore opening more likely, while pore opening worsens respiration and can increase ROS production [39,40]. Early reperfusion injury is therefore driven by interacting changes in metabolism, calcium, pH, membrane potential, and redox chemistry rather than by a simple linear ROS cascade.

Acidosis adds another layer to the process. While low pH can reduce some harmful reactions during occlusion, rapidly restoring pH after reperfusion may remove this brief protective effect. Reperfusion clears lactate and raises extracellular pH, with intracellular pH recovering through Na^+^/H^+^ exchange and bicarbonate transport. These activities can increase intracellular Na^+^ levels, potentially causing reverse Na^+^/Ca^2+^ exchange and more Ca^2+^ entry into the cell. As a result, pH normalization affects ion transport and mitochondrial permeability transition [39,40]. The quick shift from an acidic, energy-deficient state to an oxygenated, partly re-energized state is key to early reperfusion injury.

### 4.2. Neurological and Neurovascular Consequences

Early reperfusion can harm neurons that were still alive at the end of the occlusion. Some cells might recover their membrane potential but stay unstable due to damaged mitochondria, elevated intracellular calcium, and depleted antioxidant reserves. Reoxygenation may transform a partially reversible metabolic disturbance into delayed energy failure [39]. Cell death does not always happen right away; regulated death pathways can develop over the subsequent hours.

The neurovascular unit experiences early effects after reperfusion. Endothelial cells first lose ATP during ischemia, then encounter oxidative and inflammatory stress when blood flow resumes. ROS and RNS can alter junctional proteins, cytoskeletal components, enzymes, and extracellular matrix elements. Matrix metalloproteinases may activate, degrading the basal lamina and tight-junction proteins. Additionally, endothelial nitric oxide signaling can become dysregulated, and the glycocalyx may suffer damage [20,67,68,69,70,71,72]. These combined changes elevate vascular permeability and play a role in early blood–brain barrier (BBB) disruption.

Pericytes encircle capillaries and are vital for regulating microvascular diameter, supporting endothelial cell stability, and maintaining the blood–brain barrier (BBB). During ischemia, they face calcium overload, oxidative stress, ATP depletion, and contractile signals. If they stay constricted after upstream blood flow resumes, they may contribute to no-reflow phenomena. Swelling of astrocytic end-feet and endothelial cells can further compress capillaries externally. Thrombi, leukocytes, fibrin, and red blood cells can obstruct flow within the vessels [16,17,18,19,20,21,22]. This leads to uneven reperfusion, with some capillary regions recovering near-normal flow and others remaining poorly perfused.

Clinical no-reflow studies should therefore be read using their actual imaging definitions. Ng et al. and Rivet et al. evaluated tissue-level perfusion abnormalities and clinical associations, not direct capillary visualization [11,13]. In contrast, the arterial-spin-labeling study by ter Schiphorst et al. identified severe hypoperfusion with new infarction in only one of 33 patients with complete or near-complete stable recanalization [14]. Differences in imaging modality, timing, thresholds, and exclusions prevent a single prevalence estimate. A clinical perfusion deficit supports persistent hypoperfusion, while attributing it to pericytes, microthrombi, or another capillary mechanism requires additional evidence.

This uneven microvascular flow leads to two key effects. Firstly, certain tissue areas remain ischemic even if angiography shows a successful thrombectomy. Secondly, inconsistent oxygen delivery causes tissue to experience repeated fluctuations between low and higher oxygen levels. As a result, the brain may exhibit adjacent regions of ongoing ischemia, partial reperfusion, and hyperemia [13,14]. Simple cell-culture models do not accurately capture this spatial variability, which might explain why treatments targeting a single biochemical pathway often work better in experimental settings than in actual patients.

BBB injury creates a direct link between oxidative damage, edema, and hemorrhagic transformation. Damage to endothelial cells and the basement membrane leads to increased leakage of water and plasma proteins. As tight junctions weaken, vasogenic edema adds to the cytotoxic edema caused by ischemia. Reperfusion further raises hydrostatic pressure in damaged microvessels, potentially worsening swelling. Severe vascular injury can allow red blood cells to escape the vessel, resulting in hemorrhagic transformation [67,68,72,73,74,75,76]. Factors such as thrombolytic therapy, infarct size, hyperglycemia, hypertension, and delayed reperfusion can all influence this risk.

**Figure 3:**
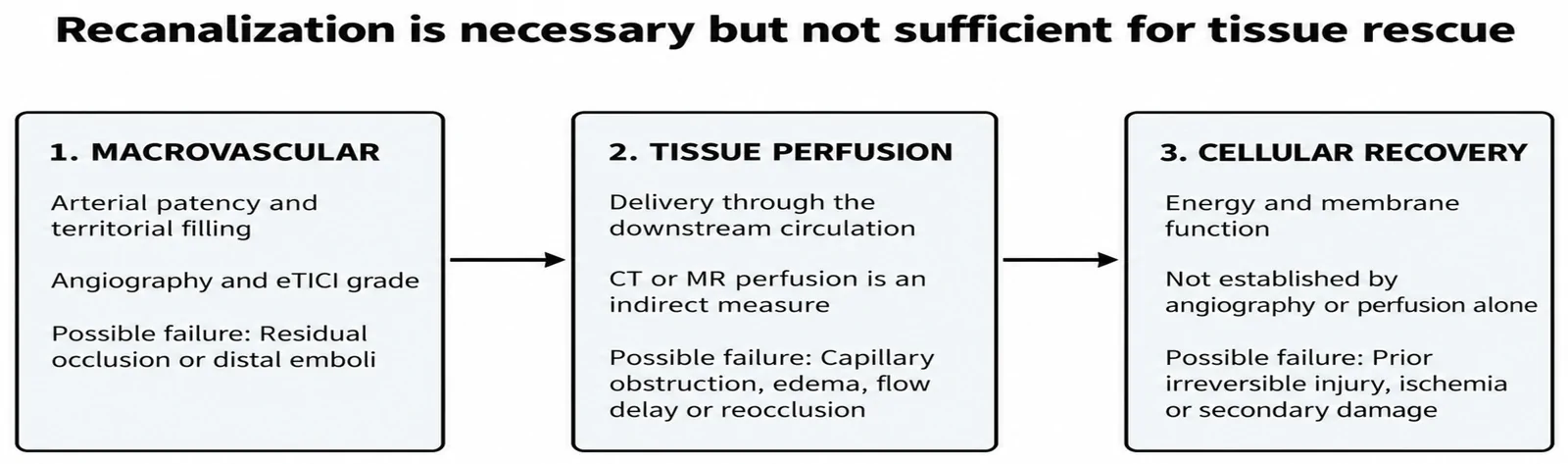
Distinct levels of recovery after thrombectomy. Angiography assesses macrovascular patency and territorial reperfusion; CT or MR perfusion assesses tissue-level delivery, while neither directly measures capillary obstruction or cellular metabolic recovery. Persistent hypoperfusion can reflect several causes, including experimental-type capillary no-reflow. Metabolic failure after reopening may reflect pre-existing irreversible injury, persistent ischemia, reoxygenation-associated damage, or secondary injury. These distinctions also affect drug delivery and interpretation of adjunctive therapies.

For patients, the key goal is effective tissue reperfusion rather than just large-vessel patency. Thrombectomy can reopen the proximal artery, but microvascular perfusion downstream may still be incomplete. Recovery happens in stages: macrovascular recanalization, microvascular reperfusion, and finally cellular metabolic recovery. Failure can occur at any stage. This is also important for neuroprotective drugs, as intravenous treatment may not evenly reach all capillary beds [11,12,13,17]. The areas most in need of protection might sometimes receive the lowest drug concentration. These three levels of reperfusion success are summarized in Figure 3.

### 4.3. Potential Neuroprotective Targets

Mitochondrial approaches might focus on targeting succinate oxidation, reversing electron transport at complex I, excessive mitochondrial ROS production, calcium uptake, or permeability transition. The goal is to temporarily reduce harmful electron flow without hindering the quick recovery of oxidative phosphorylation essential for healing [29,30,39]. Total inhibition of the respiratory chain would probably be damaging, but brief and well-timed modulation could offer protection.

Mitochondria-targeted antioxidants aim to concentrate redox-active drugs within mitochondria. However, their uptake often relies on mitochondrial membrane potential, resulting in poor accumulation in severely depolarized mitochondria during ischemia and increased accumulation only after reperfusion. This timing might be beneficial if targeting the early reoxygenation burst, but it also makes drug exposure dependent on the organelle’s condition. Radical scavengers must react quickly and reach sufficiently high local concentrations to compete with surrounding biomolecules [43,66]. Systemic antioxidant treatments frequently fail because many radicals react almost instantly; additionally, ROS play normal signaling roles, and late treatments cannot reverse already occurred oxidation.

An alternative approach is to inhibit lipid-radical chain reactions rather than trying to neutralize every initiating oxidant. Using chain-breaking antioxidants, lipoxygenase inhibitors, maintaining GPX4 activity, and stabilizing vulnerable phospholipid pools could be effective strategies. Edaravone, a free-radical scavenger used clinically in some countries, has been combined with dexborneol to enhance antioxidant and anti-inflammatory effects [31,64,65]. The clinical use of edaravone and dexborneol is detailed in Section 6.6 and is relevant for timing treatment relative to reperfusion.

Protecting the vasculature may be as crucial as safeguarding neurons. Approaches that decrease endothelial ROS, maintain the glycocalyx, relax pericytes, reduce platelet–leukocyte interactions, or limit microthrombi could enhance tissue perfusion and drug delivery [13,17,20,21,72]. While adjunct intra-arterial thrombolysis after thrombectomy is relevant, its primary goal is to improve reperfusion rather than provide direct cell protection. Some randomized studies indicate that pharmacological treatment administered via the catheter post-thrombectomy may improve outcomes in selected patients [77,78,79,80,81]. This highlights that reopening the large artery alone does not necessarily ensure complete reperfusion. Therefore, catheter-based therapies targeting the microcirculation downstream are a vital area for further research.

Distinct from reperfusion enhancement, the thrombectomy catheter could also be used to deliver cytoprotective drugs directly into the target artery, allowing high local concentrations for a brief period while reducing systemic exposure. However, this method introduces several formulation and chemistry challenges: the drug must stay stable in the infusion solution and catheter, avoid precipitation or sticking to tubing, tolerate rapid blood dilution, and not harm the endothelium at high doses. Drugs that are highly protein-bound may lose their active unbound form once in plasma. While reactive scavengers might benefit from elevated local concentrations, drugs requiring intracellular or mitochondrial uptake could have insufficient contact time before being washed out [15]. Addressing these issues should be part of drug development, ideally before efficacy is demonstrated in preclinical tests.

## 5. Injury Continuing After Reperfusion

### 5.1. Chemical and Biochemical Changes

Oxidative injury persists beyond the initial minutes of reperfusion. Early radicals can trigger chain reactions that continue even after the original oxidant is gone. Lipid peroxidation exemplifies this process: when a PUFA-containing phospholipid forms a lipid radical, oxygen addition and hydrogen transfer can propagate oxidation to neighboring lipids. These phospholipid hydroperoxides alter membrane packing, curvature, ion permeability, receptor organization, and organelle function. In mitochondria, oxidized phospholipids hinder respiration and compromise membrane stability. In the plasma membrane, they disrupt transport and signaling pathways [31,64,65]. Within endothelial cells, they can further impair barrier function.

The type of lipid being oxidized also matters. Ferroptosis research shows that acyl-CoA synthetase long-chain family member 4 (ACSL4) and lysophosphatidylcholine acyltransferase 3 (LPCAT3) can enrich phosphatidylethanolamine with arachidonic and adrenic acids, making these phospholipids particularly sensitive to peroxidation. These phospholipids can then be converted to phospholipid hydroperoxides through lipoxygenase-mediated or nonenzymatic reactions. GPX4 limits this damage by reducing membrane lipid hydroperoxides to less reactive lipid alcohols, using glutathione as the electron donor [33,65,82,83,84,85,86]. If GPX4 activity declines or glutathione is depleted, lipid peroxides can accumulate faster than the membrane can repair them.

Glutathione metabolism links peroxide defense to amino acid availability. Reduced glutathione (GSH) contains glutamate, cysteine, and glycine; glutathione reductase uses NADPH to regenerate GSH from oxidized glutathione (GSSG). Cysteine supply is cell-type dependent. System xc^−^ exchanges extracellular cystine for intracellular glutamate, whereas neuronal cysteine uptake importantly involves excitatory amino acid transporter 3 (EAAT3, also called EAAC1) [87,88]. Increasing system xc^−^ activity may support glutathione synthesis in some cells but also raise extracellular glutamate when clearance is impaired. Pharmacological inhibition or genetic impairment of this exchanger reduced oxygen–glucose-deprivation injury in experimental preparations [87]. Thus, antioxidant support and excitotoxic glutamate export must be considered together; system xc^−^ activation cannot be treated as uniformly protective in brain ischemia. ATP depletion, substrate limitation, and impaired GSH recycling may compromise GPX4-dependent peroxide removal [31,64].

Model dependence is illustrated by mice lacking functional system xc^−^: cortical injury was reduced after moderate photothrombotic ischemia but not after severe permanent middle cerebral artery occlusion [89].

Iron influences whether lipid hydroperoxides stay controlled or develop into new radical sources. The unstable Fe^2+^ pool facilitates Fenton chemistry, breaking down lipid hydroperoxides into alkoxyl and peroxyl radicals. Normally, iron homeostasis is maintained through transferrin uptake, divalent metal transporter 1 (DMT1) transport, ferritin storage, ferroportin export, heme metabolism, and iron–sulfur cluster turnover. Ferritin stores much of this iron in a relatively inactive state. Via nuclear receptor coactivator 4 (NCOA4)-mediated ferritinophagy, ferritin can be transported to lysosomes, releasing iron for cellular use. In experimental ischemia–reperfusion models, excessive ferritinophagy can increase the redox-active iron pool, promoting ferroptotic injury [32,45,90,91,92]. In oxygen-glucose deprivation/reoxygenation models, inhibiting NCOA4-dependent ferritinophagy decreases oxidative stress and cell death, indicating that targeting ferritin turnover may be beneficial.

Ferroptosis is a form of regulated cell death associated with iron-dependent phospholipid peroxidation and failure of lipid-peroxide defenses. Lipid oxidation alone is insufficient to diagnose it. Strong experimental attribution combines cell-death measurements with lipid-peroxide accumulation, iron dependence, rescue by mechanistically distinct ferroptosis inhibitors or iron chelation, and genetic perturbation of relevant regulators such as GPX4 or ACSL4 [31,64,65,82,83]. Changes in GPX4 abundance, GSH, iron, malondialdehyde (MDA), or 4-hydroxynonenal (4-HNE) are supportive but individually nonspecific. Pharmacological rescue may also reflect antioxidant effects outside ferroptosis; genetic results depend on cell type and model. Apoptosis, necroptosis, pyroptosis, necrosis, and autophagy-related processes may coexist [32,33,83,92,93,94]. The cited stroke literature provides experimental mechanistic evidence, but does not establish the proportion of human infarcted tissue dying by ferroptosis or validate a ferroptosis-specific clinical diagnostic test. Human tissue evidence would require convergent cell-resolved findings and exclusion of competing death mechanisms, rather than a single staining or circulating marker.

Lipid hydroperoxides can decompose into reactive aldehydes instead of just remaining as end products. Notable examples are 4-hydroxynonenal (4-HNE), malondialdehyde (MDA), and acrolein. These compounds are more stable than many radicals and can diffuse away from the initial oxidation site. Their carbonyl groups react with nucleophilic amino acids like cysteine, histidine, and lysine, forming covalent adducts. 4-HNE can modify proteins involved in the respiratory chain, transporters, cytoskeletal components, antioxidant enzymes, and signaling proteins. Acrolein is highly electrophilic and reacts quickly with thiols [95,96]. MDA can also create protein and nucleic acid adducts and is commonly used as a marker for lipid peroxidation, although it is not completely specific.

Reactive aldehydes have the capacity to prolong damage beyond the initial burst of ROS. A brief episode of oxidative stress can lead to lipid peroxide formation, which in turn produces aldehydes that linger and spread through tissues. These aldehydes can also impair enzymes responsible for removing them, creating a vicious cycle. Mitochondria are particularly susceptible because their membranes produce lipid-derived electrophiles and their proteins feature numerous redox-sensitive sites [95,96]. Covalent modifications of respiratory proteins can increase electron leakage and sustain ROS generation even after oxygen levels return to normal.

Aldehyde dehydrogenase 2 (ALDH2) contributes to mitochondrial aldehyde detoxification. In experimental cerebral ischemia–reperfusion, ALDH2-related interventions have reduced aldehyde burden and injury measures in animal models [76,96,97]. Reduced 4-HNE or MDA is consistent with less oxidative damage but does not independently identify the causal target. Moreover, aldehyde persistence does not demonstrate efficacy when treatment is delayed until after recanalization. Delayed-start studies, brain pharmacokinetics, and safety testing are needed before proposing a clinical window.

Cells depend on various endogenous defense mechanisms such as glutathione-S-transferases, aldo-keto reductases, superoxide dismutases, catalase, peroxiredoxins, thioredoxin systems, and the nuclear factor erythroid 2-related factor 2 (Nrf2) response. Nrf2 controls genes related to glutathione synthesis, NADPH production, quinone detoxification, heme metabolism, and protection against electrophiles. However, its response is relatively slow because it involves transcription, making Nrf2 more applicable for sustained protection and recovery rather than immediate response after recanalization [2,85,86,98]. Excessive or prolonged activation may have context-dependent effects, and any drugs targeting this pathway must reach the tissue before damage becomes irreversible.

Persistent mitochondrial dysfunction may sustain ROS production, energy failure, and inflammatory signaling. Experimental studies implicate altered fission, fusion, mitochondrial DNA release, and mitophagy; both inadequate clearance and excessive loss of mitochondrial mass can be harmful [99,100,101,102,103,104,105]. These findings concern injury continuing after reperfusion and do not establish that all such injury was caused by restoration of blood flow.

### 5.2. Neurological and Neurovascular Consequences

Neurons may die hours after reperfusion begins. Ongoing mitochondrial problems reduce ATP levels and increase cell susceptibility to secondary depolarization. Lipid peroxidation affects membrane excitability and receptor activity, while aldehyde adducts disrupt enzymes and transporters. DNA damage and redox-sensitive signals can trigger apoptosis and inflammation [32,33]. Ferroptosis could be particularly significant in cells rich in PUFA lipids, with low glutathione reserves, or poor iron regulation.

Astrocytes play a key role in both delayed injury and recovery processes. They control the levels of extracellular glutamate, K^+^, water, and metabolic substrates, and contribute to maintaining the BBB. Oxidative stress can impair glutamate transporters and disrupt aquaporin regulation. Reactive astrocytes may emit cytokines, enzymes that modify the matrix, and trophic factors. Depending on the timing and nature of their response, they can either exacerbate inflammation and edema or promote repair [2,73,74,106,107]. Therefore, maintaining astrocyte metabolism might be crucial for the long-term survival of neurons, even when neurons are directly shielded.

Oligodendrocytes and axons must also be taken into account. White matter, rich in lipids like myelin, is susceptible to lipid peroxidation. Oligodendrocytes rely on active lipid synthesis to sustain myelin and are vulnerable to oxidative stress and glutamate toxicity. Axons require mitochondrial ATP to maintain ion homeostasis and support long-distance transport. Damage to mitochondria or oxidative modifications of cytoskeletal proteins can undermine nerve conduction and harm axons beyond the visible cortical infarct [2,53,54,108]. For functional recovery after stroke, it is crucial to preserve white-matter tracts and network connections, not just neuronal cell bodies.

Barrier dysfunction may progress after recanalization through endothelial injury, inflammatory signaling, matrix metalloproteinases, and altered interactions with pericytes and astrocytes [67,68,69,70,71,72]. This can contribute to vasogenic edema and hemorrhagic transformation. Its progression provides a rationale for testing vascular protection, but does not establish a reversible treatment window or prove that reperfusion initiated the injury. Safety assessment must consider infarct extent, blood pressure, thrombolytic exposure, and concomitant antithrombotic treatment.

Neuroinflammation is intimately linked to redox injury. Damaged cells release danger signals like ATP, high-mobility group box 1 (HMGB1), mitochondrial DNA, and oxidized lipids, which activate microglia and draw in immune cells from outside. These immune cells produce more oxidants via NADPH oxidases, myeloperoxidase, nitric oxide pathways, and lipid-metabolizing enzymes. Oxidized phospholipids and aldehyde-modified proteins can also serve as inflammatory signals [17,90,94,109,110,111]. Consequently, chemical injury can initiate inflammation, which then leads to additional chemical damage.

Infarct growth can persist after successful reperfusion due to various factors. These include incomplete microvascular flow, edema, secondary thrombosis, mitochondrial dysfunction, ongoing excitotoxic signaling, lipid oxidation, ferroptosis, other cell death pathways, and inflammation. The significance of each may vary between patients [2,13,17]. Consequently, future biomarkers might be more effective if they detect the primary injury pattern specific to each individual, instead of assuming all reperfused strokes have identical molecular mechanisms.

### 5.3. Potential Neuroprotective Targets

Reactive aldehydes are candidate preclinical treatment targets because they last longer than ROS and can remain after the initial oxidative burst. Potential strategies include using aldehyde scavengers, activating ALDH2, enhancing glutathione-dependent conjugation, and maintaining the NAD^+^/NADH balance essential for aldehyde oxidation. Since aldehydes also play normal signaling roles, broad scavenging might disrupt adaptive responses [95,96,97]. Therefore, candidate drugs should be evaluated for their selectivity, reaction speed, metabolites, and the concentration required to outcompete abundant cellular nucleophiles.

Preclinical ferroptosis-directed strategies include lipid-radical trapping with ferrostatin-1 or liproxstatin-1, iron chelation, modulation of ACSL4 or LPCAT3, and support of GSH/GPX4-dependent peroxide defenses [31,32,33,64,65,82,84,86,93]. Increasing system xc^−^ activity is particularly problematic because cystine import is coupled to glutamate export; inhibition has been protective in experimental ischemia, as detailed in Section 5.1 [87]. Candidate interventions must preserve physiological iron use, membrane remodeling, and neurotransmitter homeostasis. Brain exposure, cell specificity, and interactions with thrombolysis require testing.

A later window for ferroptosis- or aldehyde-directed treatment remains a hypothesis. Evidence that lipid peroxidation or aldehydes persist for hours does not show that cells are still salvageable or that delayed drug delivery can alter outcome [32,33,96]. Studies should compare prespecified treatment starts before, at, and after reperfusion, measure free drug exposure in relevant compartments, and assess durable functional benefit. Pretreatment or treatment at reperfusion cannot substantiate efficacy several hours later.

Vascular and glial targets include endothelial junctions, pericyte–endothelial interactions, matrix metalloproteinases, astrocyte metabolism, and oligodendrocyte lipid regulation. A sequence combining early metabolic support with later lipid-, barrier-, or inflammation-directed treatment is a research hypothesis [2,15]. Section 6.7 sets out the additional safety and design requirements.

## 6. Translation into the Thrombectomy Era

Neutral neuroprotection trials may reflect an ineffective target or intervention, inadequate brain exposure, treatment after irreversible injury, or a mismatch between the selected population and the proposed mechanism [15,23,24,25,26,27]. The available data do not justify attributing all failures to timing or delivery. Even a well-delivered drug may have an insufficient effect, adverse off-target actions, or no clinically relevant effect on the pathway. Mechanistic explanations therefore require prospectively collected evidence rather than retrospective rescue of a negative efficacy result.

Thrombectomy alters this scenario in three key ways: (1) it enables rapid reperfusion that can be verified angiographically [4,5,7,8]; (2) it provides a defined reperfusion time point around which drug administration can be coordinated; and (3) the endovascular catheter provides direct access to the affected arterial circulation [15]. While these advantages do not ensure the success of neuroprotection, they facilitate clinical trials that investigate biological mechanisms with substantially improved control of treatment timing.

### 6.1. Treatment Timing as a Mechanistic Variable

Treatment timing is a mechanistic hypothesis that must be tested for each candidate. Energy-preserving or anti-excitotoxic approaches may need exposure during occlusion, and inhibitors of an early reoxygenation-associated process may need active concentrations before flow restoration [29,30,33]. Ferroptosis, aldehyde formation, barrier failure, and inflammation overlap these intervals; their persistence does not establish that treatment started later remains effective. The framework therefore specifies candidate exposure periods, not a validated therapeutic timetable.

This timing difference directly impacts pharmacokinetics. When the therapeutic window is only minutes, the terminal plasma half-life matters less than how fast the drug attains an active free concentration in the ischemic neurovascular unit. A long-acting, slow-distributing drug may be unsuitable for early reperfusion injury, while a short-acting drug might be more effective if it quickly reaches its target and the harmful reaction is brief [15]. Trials should record the time from drug administration to reperfusion and, when possible, include measured plasma or tissue levels instead of relying solely on onset-to-treatment time.

Reperfusion-linked treatment is operationally attractive but difficult to synchronize precisely. Vessel reopening may occur unexpectedly on the first pass, and angiographic reperfusion does not establish simultaneous capillary reflow. Intravenous administration before anticipated reperfusion may improve readiness but depends on collateral delivery; intra-arterial administration after a successful pass may miss very early reactions. Both routes require rapid formulation, acceptable local and systemic exposure, and compatibility with the procedure [15,30]. No investigational adjunct should delay established reperfusion therapy.

### 6.2. Patient Selection Using Imaging, Penumbra, and Collateral Status

Core size, clinical–imaging mismatch, collateral status, and perfusion imaging have demonstrated value in selecting or characterizing populations for thrombectomy [46,47,48,49,50,51,52]. They are not validated measures of preserved mitochondrial function, GSH/GPX4 capacity, or ferroptosis susceptibility. A small core and substantial penumbra indicate potentially salvageable tissue but do not identify a specific biochemical target. Associations between these imaging states and mechanism-specific treatment responsiveness require prospective validation.

Future studies may stratify participants by baseline tissue status, occlusion site, collateral grade, and achieved reperfusion. Such stratification can test whether treatment effects differ across clinically defined groups [46,47]. It should not be described as molecular enrichment unless an imaging or blood marker has been validated against the relevant pathway. Persistent hypoperfusion may be useful for trials of reperfusion enhancement, after alternative causes such as residual occlusion or reocclusion are evaluated.

Collateral flow and residual microvascular dysfunction can influence drug distribution, but perfusion imaging does not directly measure brain drug concentration [13,46,47]. Intravenous agents may reach threatened tissue through collaterals; local arterial delivery can still be limited by downstream flow failure and intracellular uptake. Serial imaging can characterize perfusion and infarct evolution, while pharmacokinetic sampling and target-specific assays are needed to assess exposure and engagement.

### 6.3. Treatment Before, During, or Immediately After Reperfusion

Administering treatment before reperfusion offers two key benefits: it may preserve tissue during occlusion and is on board when oxygen flow resumes. Clinical testing requires systemic drugs that have a high safety margin and do not reduce blood pressure or hinder thrombolysis or anesthesia. These medications could be initiated in the ambulance or at an initial hospital if patient eligibility is straightforward [15]. However, a primary limitation is diagnostic uncertainty, as some patients who receive treatment might not ultimately require thrombectomy.

Thrombectomy treatment enables more precise timing, as IV access and monitoring are already in place, allowing operators to anticipate reperfusion timing. A bolus can be aligned with the final device pass, and intra-arterial delivery becomes feasible. However, since thrombectomy is highly time-sensitive, a neuroprotective protocol must not delay arterial puncture, clot removal, or reperfusion [15]. Therefore, drug preparation should be quick, straightforward, and seamlessly integrated into the standard angiography workflow.

Post-reperfusion administration allows selection according to the achieved angiographic result and procedural complications. It is a feasible workflow for testing lipid-, aldehyde-, barrier-, or inflammation-directed candidates, but feasibility is not evidence of efficacy [32,33]. Treatment may already be too late for rapidly irreversible reactions. Delayed-start experiments and exposure–response data should determine which candidates merit clinical testing after recanalization.

### 6.4. Systemic Versus Intra-Arterial Administration

Intravenous treatment is well-known, scalable, and often feasible to initiate before arriving at the thrombectomy center. Its main challenge lies in distribution: the drug disperses through the body, may bind tightly to plasma proteins, be absorbed by other organs, and may poorly reach severely hypoperfused brain tissue. For compounds with high protein binding, total plasma levels might overstate the active free drug. Additionally, rapid liver metabolism may necessitate higher doses for sufficient brain exposure, raising systemic toxicity risks [15]. Variability in blood–brain barrier (BBB) permeability plays a role too, as it can remain relatively intact in some penumbral areas before reperfusion and become compromised in others afterward.

Intra-arterial delivery can achieve significantly higher short-term concentrations in the affected circulation. This approach may be advantageous for short-lived scavengers, reversible enzyme inhibitors, or drugs that cannot be administered at high systemic doses. However, a high local concentration does not guarantee that an intracellular target will be reached. Lipophilic drugs tend to quickly enter membranes, while hydrophilic compounds may mostly stay within the vascular space unless facilitated by transporters or BBB disruption. Peptides can be degraded or bind to plasma proteins, and highly reactive compounds might be consumed before reaching the target tissue [15]. Additionally, the formulation must remain compatible with contrast media, heparinized saline, catheter materials, and the ionic conditions of blood.

Medicinal chemistry considerations should be addressed early for every intra-arterial candidate. Is the molecule stable during preparation and infusion? Is it soluble at the required concentration for local arterial delivery? Does it adsorb to plastic tubing? Is it stable at physiological pH and in plasma? What fraction remains unbound? Does albumin binding help maintain a drug reservoir or prevent tissue entry? How quickly does the molecule cross the BBB before and after reperfusion? Does it require active transport? Are its metabolites active or toxic? Can it work as a bolus, or is a longer infusion necessary to sustain target occupancy [15,112]? These practical properties influence whether a convincing biological mechanism can be effectively tested in patients.

### 6.5. Interactions with Thrombolytic Therapy

Any neuroprotective drug used in thrombectomy patients must be compatible with alteplase and tenecteplase, as many eligible patients still undergo bridging thrombolysis. Interactions can be pharmacodynamic, affecting bleeding or platelet function, or pharmacokinetic, such as degradation of a peptide by proteolytic enzymes. Nerinetide serves as a good example. Although ESCAPE-NA1 did not achieve its primary endpoint, an exploratory analysis indicated potential benefits for patients not treated with alteplase. Exposure to alteplase was linked to reduced nerinetide levels, likely because plasmin cleaved the peptide [59]. This finding prompted the ESCAPE-NEXT trial, which evaluated nerinetide in thrombectomy patients who had not received intravenous thrombolysis.

ESCAPE-NEXT did not show benefit despite excluding previous intravenous thrombolysis: mRS 0–2 at 90 days occurred in 45% versus 46%, with an odds ratio of 0.97 (95% CI 0.72–1.30) [113]. This weakens the explanation that the alteplase interaction alone accounts for the earlier neutral result. It does not prove that excitotoxicity is irrelevant, nor establish inadequate exposure as the explanation. Plasma pharmacokinetics can identify systemic exposure, but adequate plasma levels alone do not establish delivery to threatened brain or engagement of PSD-95-dependent signaling.

Small molecules are less susceptible than peptides to proteolytic breakdown, but they can still influence processes like hemostasis, endothelial function, liver metabolism, or renal clearance. Antioxidants might modify platelet redox signaling, iron chelators could interact with coagulation proteins, and drugs affecting nitric oxide signaling may alter bleeding risk. Compatibility testing should address both pharmacokinetic and pharmacodynamic interactions. Peptide degradation, altered platelet or coagulation function, effects on fibrinolysis, endothelial permeability, and systemic blood pressure may affect benefit or hemorrhagic risk [59,113]. Candidate-specific testing with alteplase or tenecteplase is necessary; compatibility with one co-treatment cannot be assumed for another. Trials should prespecify symptomatic intracranial hemorrhage, any intracranial hemorrhage, systemic bleeding, edema, mortality, and organ toxicity. Apparent protection of a molecular barrier marker is not evidence of clinical hemorrhagic safety.

### 6.6. Clinical Studies and What They Teach

The neutral ESCAPE-NA1 and ESCAPE-NEXT results illustrate the distinction between an unsuccessful regimen and a disproven biological pathway [59,113]. Potential explanations include inadequate target engagement, insufficient tissue penetration or dose, treatment after irreversible injury, patient heterogeneity, and weak target relevance in the enrolled population. Off-target effects may counterbalance benefit. Outcome selection also matters, but changing endpoints after a neutral trial cannot establish efficacy. Prospective exposure measurements, pathway-specific pharmacodynamic measures, prespecified timing strata, and clinically meaningful functional endpoints are needed to distinguish these explanations. Without such evidence, timing, dose, and delivery remain possibilities rather than demonstrated reasons for failure.

For edaravone dexborneol, the 200-patient phase II study reported 90-day mRS 0–2 in 58.7% versus 52.1% of participants with outcome data, with a reported unadjusted odds ratio of 1.37 (95% CI 0.76–2.44); the primary endpoint was neutral [114]. In TASTE-2, 1362 patients were randomized and 1360 contributed to the efficacy analysis. Functional independence occurred in 55.0% versus 49.6% (risk ratio 1.11, 95% CI 1.00–1.23; risk difference 5.4 percentage points, 95% CI 0.1–10.7; *p* = 0.05). Serious adverse events occurred in 27.2% versus 25.7% [115]. The borderline overall estimate and subgroup findings require cautious interpretation. Differences in sample size and dosing relative to thrombectomy may contribute to the contrasting results, but these trials do not isolate timing as a causal modifier or validate a specific ferroptosis mechanism.

Adjunctive intra-arterial thrombolysis is a reperfusion-enhancing strategy rather than a neuroprotective intervention, as its primary objective is to restore downstream blood flow rather than directly prevent biochemical injury associated with reperfusion. CHOICE was positive but stopped early; POST-TNK was neutral; ANGEL-TNK and PEARL reported positive primary functional endpoints in selected populations [77,78,79,80]. CHOICE-2 also favored alteplase for excellent functional outcome and reduced CT-perfusion hypoperfusion, but mortality was higher with alteplase [81]. These results do not establish that capillary no-reflow caused the deficits or that the drugs directly inhibited reoxygenation injury. Perfusion-imaging improvement is a surrogate; functional outcome, hemorrhage, and mortality require separate assessment.

The randomized trials test distinct interventions and should not be treated as evidence for a single class effect. Direct cytoprotection aims to reduce cellular injury, whereas reperfusion enhancement improves oxygen and substrate delivery; oxygen supplementation represents a further distinct strategy [56,59,77,78,79,80,81,113,114,115]. Table 2 separates these categories and reports eligibility, intravenous thrombolysis exposure, achieved reperfusion requirements, timing, primary effects with confidence intervals, and safety findings. Positive or neutral classification refers to the prespecified primary endpoint, not to mechanistic proof or readiness for routine practice.

Further randomized trials illustrate the heterogeneity of adjunctive intra-arterial thrombolysis. POST-UK did not significantly improve 90-day mRS 0–1 after near-complete or complete reperfusion (adjusted RR 1.13, 95% CI 0.94–1.36) [116]. Posterior-circulation trials were also neutral: ATTENTION-IA reported an adjusted RR of 1.36 (95% CI 0.92–2.02) for mRS 0–1 with tenecteplase, with a numerically higher sICH rate (8.3% versus 3.1%); IAT-TOP reported an adjusted RR of 0.93 (95% CI 0.73–1.18) for mRS 0–2 with alteplase [117,118]. These results further argue against treating adjunctive thrombolysis as a uniform class effect or extrapolating between vascular territories. Table 2 presents selected trials, not an exhaustive catalogue.

### 6.7. Future Thrombectomy-Based Trial Designs

Future trials should prespecify treatment timing relative to tissue reperfusion, document dose and infusion completion, and measure pharmacokinetic exposure and candidate-specific target engagement. Baseline imaging can support established clinical selection and prespecified stratification, while molecular enrichment should remain investigational until validated. Primary efficacy outcomes should assess clinically meaningful disability, usually the 90-day mRS, with mortality reported separately. Infarct volume, perfusion parameters, early neurological change, and biochemical measures are useful mechanistic or intermediate outcomes, but do not substitute for durable functional benefit [15,23,24,30,59,115].

**Figure 4:**
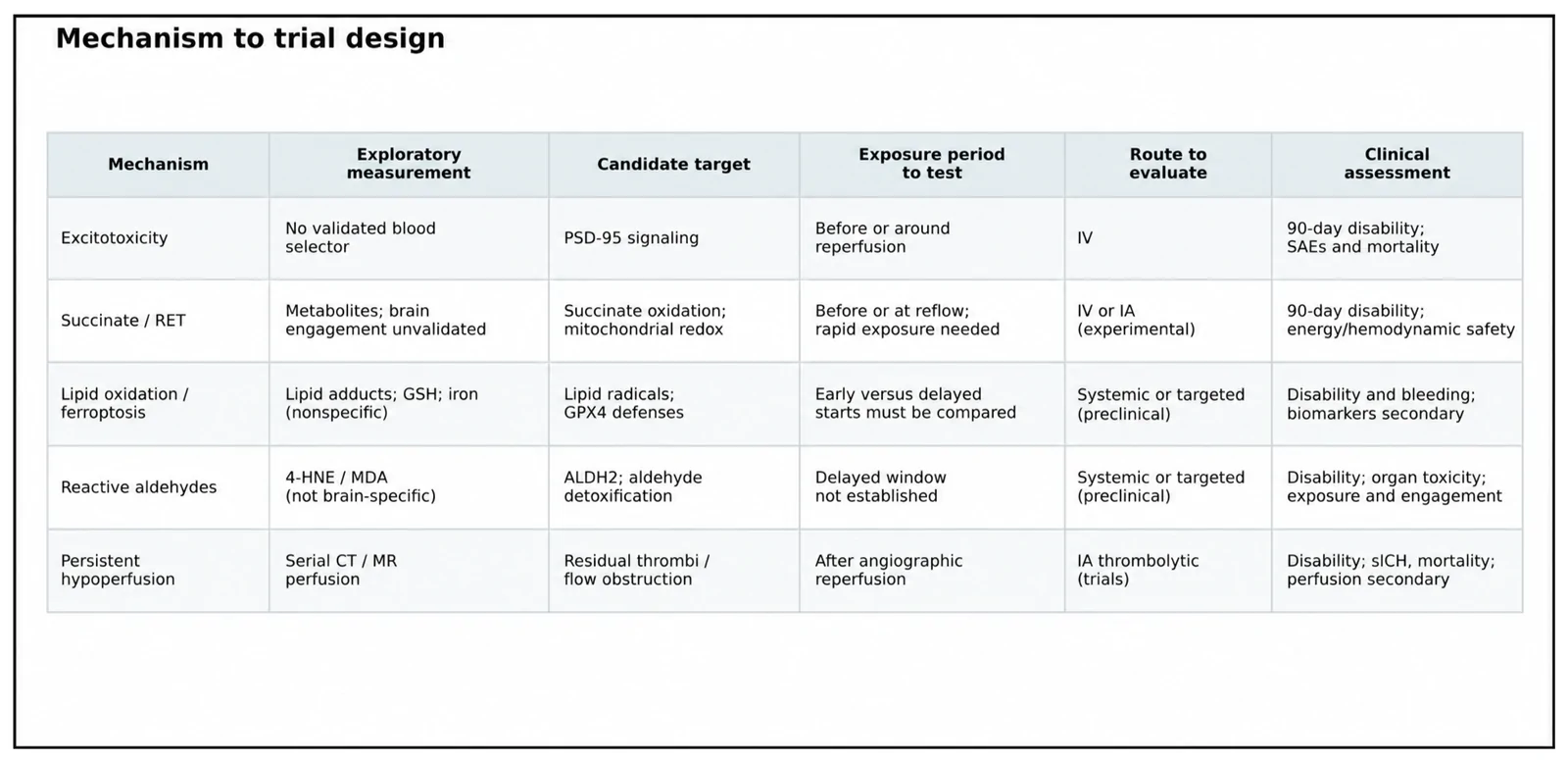
Candidate links from mechanism to measurement, treatment exposure, route, and clinical assessment. Biomarkers and proposed exposure periods are exploratory. The diagram does not validate molecular patient selection or a delayed-treatment window. IA, intra-arterial; IV, intravenous; MDA, malondialdehyde; RET, reverse electron transport; SAE, serious adverse event; sICH, symptomatic intracranial hemorrhage.

Exploratory biomarker categories include systemic oxidative-stress measures; lipid-peroxidation products such as 4-HNE adducts and MDA; GSH/GSSG and GPX4-related assays; ferritin and other iron-handling proteins; mitochondrial injury markers, including circulating mitochondrial DNA; inflammatory mediators; cell-free DNA; and imaging-defined persistent hypoperfusion [13,32,33,96]. These measures differ in tissue origin, specificity, and analytical stability. Hemolysis, peripheral inflammation, comorbid disease, renal clearance, and sample processing can confound circulating values. Plasma measurements alone cannot diagnose cerebral ferroptosis or prove engagement of a brain target. A marker of established injury is not necessarily predictive of treatment response. Prospective validation requires prespecified sampling, analytical reproducibility, evidence linking the marker to the relevant cerebral process, and a treatment-by-marker interaction before use for treatment selection. Drug concentration measures exposure; a validated pathway assay is needed for engagement. Figure 4 links candidate mechanisms to exploratory measurements and trial requirements.

Adaptive or biomarker-enriched designs should distinguish exploratory discovery from confirmatory testing. A perfusion-defined subgroup may be appropriate for testing reperfusion enhancement, but blood iron or lipid-oxidation markers should not currently serve as validated selectors for ferroptosis-directed therapy [13,46,47]. Thrombolysis, onset-to-reperfusion interval, collateral grade, and core size should be prespecified covariates or stratification factors. Subgroup analyses require interaction tests and control of multiplicity; an apparently favorable subgroup after a neutral primary outcome remains hypothesis-generating.

Phased combination therapy remains an unvalidated research hypothesis. Combining metabolic, mitochondrial, vascular, and lipid-directed drugs introduces pharmacokinetic and pharmacodynamic interactions, bleeding risk, cumulative organ toxicity, and practical adherence or treatment-completion problems [2,15]. Preclinical and early-phase studies should assess each component and the combination under relevant thrombolytic exposure. Factorial or staged designs may help distinguish component effects, but require adequate power, prespecified interactions, a limited endpoint hierarchy, and multiplicity control. Concurrent biomarker changes cannot by themselves identify which pathway caused any clinical benefit.

Translation requires an exposure profile that is compatible with both the proposed biology and the thrombectomy workflow. Stability, solubility, unbound concentration, tissue distribution, intracellular uptake, target residence time, and elimination should be established before efficacy trials [15,23,24,30,112]. Serial target-engagement measures and delayed-start experiments are particularly important where a treatment window is inferred from persistent pathway activity.

### 6.8. Limitations of Translating Experimental Mechanisms

Experimental cerebral ischemia does not fully reproduce human large-vessel occlusion. Species differ in cerebrovascular anatomy, collateral networks, white-matter proportion, mitochondrial biology, immune responses, and blood–brain barrier properties. Young healthy animals, often of one sex, do not capture the effects of aging, sex, hypertension, diabetes, atherosclerosis, and multimorbidity. Anesthesia and temperature control may alter metabolism and injury; imposed occlusion duration and abrupt reperfusion differ from uncertain onset, variable infarct size, multiple thrombectomy passes, residual emboli, and heterogeneous tissue reflow in patients [23,24,25,26,27,28].

Drug effects also depend on thrombolytic exposure, antithrombotic co-treatment, protein binding, organ clearance, species-specific metabolism, and tissue distribution. Oxygen–glucose deprivation and reoxygenation cultures lack collateral flow, circulating immune cells, and intact barrier and vascular interactions. Human ex vivo ischemic brain tissue provides evidence in human material but not evidence of in vivo target dominance or therapeutic efficacy [30]. For succinate/RET, lipid-peroxide/GPX4, ferritinophagy, and ALDH2 approaches, independent replication, genetic and pharmacological convergence, delayed treatment, clinically relevant comorbid models, and durable behavioral outcomes should precede confident clinical extrapolation [29,30,31,32,33,64,76,96,97].

Hemorrhagic transformation is a cross-cutting constraint. For mitochondrial and redox candidates, systemic hemodynamics, energy production, and platelet effects require evaluation; for iron- and lipid-directed agents, physiological iron use, membrane function, and hemostasis must be preserved. Barrier-directed agents need direct hemorrhage and edema outcomes, and fibrinolytic adjuncts need particular scrutiny after IVT or antithrombotics. The higher mortality in CHOICE-2 illustrates why better functional or perfusion endpoints cannot replace an independent safety assessment [81]. Neutral hemorrhage comparisons in modest samples do not exclude clinically important harm.

## 7. Conclusions and Future Perspectives

Thrombectomy has opened significant opportunities for neuroprotection but has also clarified ongoing biological challenges. Achieving reliable reopening of the large artery eliminates one uncertainty source, yet tissue reperfusion and biochemical recovery can still fail downstream. A phase-based approach helps link these mechanisms to treatment strategies. During occlusion, energy failure and calcium overload are key issues. Early reperfusion triggers rapid redox changes, mitochondrial electron flow alterations, ROS and RNS formation, iron-dependent reactions, and lipid oxidation. Subsequent injury phases involve phospholipid hydroperoxides, reactive aldehydes, ferroptosis, ongoing mitochondrial dysfunction, BBB disruption, and inflammation [2,13,33,81,112]. Matching treatment timing to the phase in which a target is most active is a biologically plausible, testable strategy for improving tissue recovery and neurological outcomes after successful recanalization, but its clinical benefit remains to be established.

Future translational and clinical studies should test this phase-based framework by matching drug chemistry and pharmacokinetics to the biological phase being targeted. The most informative thrombectomy-era trials should therefore prespecify treatment timing relative to documented reperfusion, incorporate measures of microvascular and tissue reperfusion in addition to angiographic recanalization, and include mechanistic biomarkers whenever feasible. Imaging-based patient selection should identify salvageable tissue or residual hypoperfusion, while biological enrichment should prioritize patients with evidence of the molecular pathway being targeted, where reliable biomarkers are available. Trials should also assess pharmacokinetic exposure and target engagement and evaluate clinically meaningful endpoints, including 90-day functional outcomes and prespecified safety measures. Treatment timing should be evaluated for each candidate in relation to tissue viability, pathway activity, and pharmacokinetic exposure. Strategies aimed at preserving metabolic reserve depend on the presence of salvageable tissue, whereas interventions targeting reoxygenation-associated ROS generation may require adequate exposure before or during tissue reperfusion. Persistent hypoperfusion and lipid-, aldehyde-, barrier-, and inflammation-related injury represent potential targets after recanalization, but their persistence does not establish that treatment initiated later remains effective. The efficacy and safety of such delayed interventions must therefore be established through delayed-treatment experiments and prospective clinical trials. Combination regimens are biologically attractive but should be assembled from agents with non-overlapping toxicity and demonstrable target engagement [13,15,30,33,81,112]. The central translational objective is not simply to add a neuroprotective drug to thrombectomy, but to define which molecular process remains reversible in which patient, at which time, and by which route of delivery.

## Figures and Tables

**Table 1:** Overlapping temporal phases of ischemia and reperfusion linking dominant chemistry to neurovascular consequences and candidate therapeutic windows.

Phase	Approximate Window	Dominant Biochemical State	Principal Consequences	Candidate Therapeutic Approaches
Ischemia	Occlusion to recanalization, may persist regionally after recanalization	ATP depletion; acidosis; ionic failure; glutamate/Ca^2+^ overload; NADH and succinate accumulation; iron/lipid priming	Anoxic depolarization; cytotoxic edema; excitotoxic injury; progressive penumbral failure	Preserve penumbra; reduce energy demand/excitotoxicity; maintain mitochondrial and collateral function
Early reperfusion	Seconds to first minutes after flow restoration, timing varies spatially	Succinate oxidation; reverse electron transport; ROS/RNS burst; pH normalization; Fenton chemistry; lipid-radical initiation; permeability transition	Delayed energy failure; endothelial injury; pericyte constriction; heterogeneous capillary flow/no-reflow; early BBB disruption	Transiently modulate mitochondria/redox chemistry; protect microcirculation
Injury continuing after reperfusion	Minutes to hours/days, processes can start during ischemia or early reflow	Phospholipid hydroperoxides; reactive aldehydes; GSH/GPX4 failure; ferroptosis; mitochondrial quality-control failure; inflammation	Vasogenic edema; hemorrhagic transformation; delayed neuronal/glial/white-matter injury; infarct growth	Target lipid-radical propagation, iron/ferritinophagy, aldehydes, BBB, glia and inflammation; consider combination therapy

**Table 2:** Selected randomized thrombectomy-adjunct trials classified by therapeutic strategy and primary endpoint.

Trial and Principal Eligibility	Intervention and Reperfusion Timing	Primary Endpoint and Effect	Principal Safety Findings
ESCAPE-NA1 (2020) [59] PSD-95 cytoprotection n = 1105; disabling LVO ≤ 12 h; ASPECTS > 4; moderate/good collaterals; IVT allowed.	Nerinetide 2.6 mg/kg IV during EVT; randomized before the final reperfusion result; no achieved eTICI threshold for enrollment.	90-day mRS 0–2: 61.4% vs. 59.2%; adjusted RR 1.04 (95% CI 0.96–1.14). Neutral overall.	SAEs similar overall. Alteplase-associated reduction in drug exposure motivated further testing; subgroup findings do not establish efficacy.
ESCAPE-NEXT (2025) [113] PSD-95 cytoprotection n = 850; anterior LVO ≤ 12 h; ASPECTS > 4; prior IVT excluded.	Nerinetide 2.6 mg/kg IV in the EVT workflow; achieved reperfusion not an enrollment requirement.	90-day mRS 0–2: 45% vs. 46%; OR 0.97 (95% CI 0.72–1.30). Neutral.	SAEs occurred equally between groups; no efficacy despite exclusion of prior thrombolysis.
OPENS-2 (2025) [56] Oxygen supplementation n = 282; anterior LVO ≤ 6 h; age 18–80; EVT candidates; IVT allowed.	100% oxygen at 10 L/min for 4 h, or FiO_2_ 1.0 if intubated; begun in the EVT workflow; no achieved reperfusion prerequisite.	90-day ordinal mRS: adjusted common OR 1.65 (95% CI 1.09–2.50). Positive.	90-day mortality: 10% vs. 12%; SAEs: 20% vs. 23%. No statistically significant safety difference.
Late-window NBO phase IIb (2026) [57] Oxygen supplementation n = 120; anterior LVO; 6–24 h; age ≥ 18; IVT included in adjusted analyses.	100% oxygen at 10 L/min for 4 h, starting before recanalization; EVT planned, without an achieved reperfusion prerequisite.	≥30% NIHSS reduction at 24 h: adjusted OR 2.86 (95% CI 1.12–7.45). Positive early endpoint; 90-day mRS shift neutral (OR 1.52, 0.87–2.63).	Mortality and sICH did not differ significantly. Small phase IIb trial; early neurological improvement is not equivalent to durable disability benefit.
Edaravone dexborneol phase II (2025) [114] Antioxidant/anti-inflammatory cytoprotection 200 analyzed; age 18–80; anterior LVO ≤ 9 h; NIHSS ≥ 6; prestroke mRS ≤ 1; IVT allowed.	37.5 mg IV twice daily for 12 days; first dose within 60 min after mTICI 2b–3 reperfusion.	90-day mRS 0–2: reported unadjusted OR 1.37 (95% CI 0.76–2.44). Neutral.	sICH at 48 h: 5.3% vs. 10.9%; OR 0.46 (95% CI 0.15–1.37). Safety superiority not established.
TASTE-2 (2026) [115] Antioxidant/anti-inflammatory cytoprotection n = 1362; anterior LVO ≤ 24 h; age 18–80; NIHSS 6–25; ASPECTS 6–10; IVT allowed.	37.5 mg IV before EVT, then twice daily for 10–14 days; no achieved reperfusion prerequisite at randomization.	90-day mRS 0–2: RR 1.11 (95% CI 1.00–1.23); RD 5.4 (0.1–10.7). Borderline positive (*p* = 0.05).	SAEs: 27.2% vs. 25.7%; RR 1.06 (95% CI 0.89–1.26). Generalization beyond the enrolled population requires caution.
CHOICE (2022) [77] Reperfusion enhancement n = 121 randomized, 113 treated as randomized; LVO ≤ 24 h; IVT allowed.	Alteplase 0.225 mg/kg IA (maximum 22.5 mg), over 15–30 min after eTICI 2b50–3.	90-day mRS 0–1: adjusted RD 18.4 (95% CI 0.3–36.4). Positive; stopped early.	sICH at 24 h: 0% vs. 3.8%; mortality at 90 days: 8% vs. 15%. Limited precision; premature termination.
POST-TNK (2025) [78] Reperfusion enhancement n = 540; proximal anterior LVO ≤ 24 h; prior IVT excluded.	Tenecteplase 0.0625 mg/kg IA after eTICI 2c–3 reperfusion.	90-day mRS 0–1: adjusted RR 1.15 (95% CI 0.97–1.36). Neutral.	sICH at 48 h: 6.3% vs. 4.4%; adjusted RR 1.43 (0.68–2.99). Mortality: 16.0% vs. 19.3%; any intracranial hemorrhage increased.
ANGEL-TNK (2025) [79] Reperfusion enhancement n = 256 randomized, 255 analyzed; anterior LVO; 4.5–24 h; prior IVT excluded.	Tenecteplase 0.125 mg/kg IA after eTICI 2b–3 reperfusion.	90-day mRS 0–1: RR 1.44 (95% CI 1.06–1.95). Positive; secondary efficacy endpoints did not corroborate the primary finding.	sICH at 48 h: 5.6% vs. 6.2%; RR 0.95 (0.36–2.53). Mortality: 21.4% vs. 21.7%.
PEARL (2025) [80] Reperfusion enhancement n = 324; anterior LVO ≤ 24 h; guideline-based IVT allowed.	Alteplase 0.225 mg/kg IA (maximum 20 mg) after eTICI ≥ 2b50.	90-day mRS 0–1: adjusted RR 1.45 (95% CI 1.08–1.96). Positive.	sICH at 36 h: 4.3% vs. 5.0%; mortality: 17.1% vs. 11.3%. Numerical mortality increase was not statistically significant.
CHOICE-2 (2026) [81] Reperfusion enhancement n = 440 randomized; LVO ≤ 24 h; IVT allowed.	Alteplase 0.225 mg/kg IA (maximum 20 mg), over 15 min after eTICI 2b50–3.	90-day mRS 0–1: adjusted RD 15.0 (95% CI 5.7–24.3). Positive; reduced residual CT-perfusion hypoperfusion.	sICH: 1.4% vs. 0.5%. Mortality: 12.1% vs. 6.4%; adjusted RD 5.9 (95% CI 0.5–11.3). Mortality signal requires further study.

Abbreviations: ASPECTS, Alberta Stroke Program Early CT Score; BID, twice daily; CI, confidence interval; eTICI, expanded Treatment in Cerebral Ischemia; EVT, endovascular thrombectomy; FiO_2_, fraction of inspired oxygen; IA, intra-arterial; IV, intravenous; IVT, intravenous thrombolysis; LVO, large-vessel occlusion; mRS, modified Rankin Scale; mTICI, modified Treatment in Cerebral Ischemia; n, number randomized unless specified otherwise; NBO, normobaric hyperoxia; NIHSS, National Institutes of Health Stroke Scale; OR, odds ratio; PSD-95, postsynaptic density protein 95; RD, risk difference; RR, risk ratio; SAE, serious adverse event; sICH, symptomatic intracranial hemorrhage. Effects compare intervention with control; RD values are percentage points. Eligibility summaries are selective, not full protocols. Primary-endpoint classification is descriptive; neutral means no statistically significant superiority, not equivalence or proof of no effect. Safety comparisons are not evidence of equivalence. Trial-specific reperfusion grades and hemorrhage definitions are retained. For the edaravone phase II trial, 204 were initially randomized, and 200 entered the reported modified intention-to-treat cohort after four post-randomization exclusions. TASTE-2 allowed alteplase but excluded prior IV thrombolytics other than alteplase.

## Data Availability

No new data were created or analyzed in this study. Data sharing is not applicable to this article.
